# Experiences of pregnancy and motherhood among women with disabilities in the district of Nkangala, Mpumalanga province

**DOI:** 10.4102/ajod.v14i0.1765

**Published:** 2025-12-12

**Authors:** Maritta Zinhle, Mygirl P. Lowane, Thembi V. Simbeni

**Affiliations:** 1 Department of Public Health, School of Health Care Sciences, Sefako Makgatho Health Sciences University, Pretoria, South Africa

**Keywords:** pregnancy, women, motherhood, disabilities, sexual reproductive health, social integration

## Abstract

**Background:**

Experiences of pregnancy and motherhood needs among women with disabilities are not often discussed by families, communities, and even within healthcare facilities, resulting in their reproductive health needs being overlooked and inadequately supported.

**Objectives:**

This study aimed to explore experiences of pregnancy and motherhood among women with disabilities within Nkangala District, Mpumalanga province.

**Method:**

A qualitative, exploratory descriptive design was used among a sample of 10 women with disabilities who met the inclusion criteria. Snowball sampling method was used to recruit participants, and the sample size was determined by data saturation. One-on-one in-depth interviews using an unstructured interview guide were used to collect data.

**Results:**

Analysis revealed seven major themes reflecting women’s experiences: unplanned pregnancy; mixed emotions including relief; decisions around pregnancy termination; support received during pregnancy and postpartum; attitudes of community members and healthcare professionals; difficulties in fulfilling motherly roles; and challenges of raising a child while with a disability. Women who received consistent maternal care and strong family support were able to nurture and care effectively for their children.

**Conclusion:**

The healthcare sector needs to improve policies related to the reproductive health of women with disabilities, how they are treated to reduce the stigma and the negative comments that healthcare workers make to this population group during pregnancy and motherhood.

**Contribution:**

The study contributes to a deeper understanding of disability, promotes inclusion and challenges harmful conceptions. This knowledge can eventually improve maternal care, improve the results for mothers and children, and increase the social integration for women with disabilities.

## Introduction

The Global Report on Health Equity for Persons with Disabilities estimates that the number of people with some form of disability is more than 1.3 billion, representing about 16% of the world’s population (Pita et al. 2023). According to a global health survey, women with disabilities are 60% higher than men living with disabilities, and most of them are presenting with physical, mental, intellectual, and sensory disabilities (Matin et al. 2021). Because disabilities vary widely in type and complexity, the term disability is used differently across multiple contexts, such as legal frameworks, policy documents, clinical and educational settings, and everyday language (Goldiner 2022). As a result, it serves multiple, and sometimes contested, functions, including guiding anti-discrimination classifications, determining eligibility for various benefits, and informing decisions about the provision of specific treatments (Gillies 2024).

Despite widespread use of disability definitions, the term has no universally accepted meaning (Goldiner 2022). However, Jaiswal et al. (2024) indicated that the World Health Organization (WHO) has developed the International Classification of Functioning, Disability and Health (ICF). The ICF is a standardised framework for describing and measuring health and disability. It was adopted in 2001 by 191 WHO Member States and became an international standard for describing ability, disability and measuring the performance in the following domains:

- *Impairments* are problems in body function or alterations in body structure (e.g. paralysis, blindness, loss of a limb or memory loss).
- *Activity limitations* are difficulties in executing activities (e.g. walking, eating, difficulty seeing, hearing or problem solving).
- *Participation restrictions* are problems with involvement in any area of life (e.g. facing discrimination in employment, transportation, engaging in social and recreational activities and obtaining health care and preventative services).

Consequently, despite the different perspectives used to define disability, persons with disabilities generally share similar characteristics, namely, having functional limitations that may be affected or exacerbated by social, environmental, or human-made barriers (Luyen 2022). Access to sexual and reproductive health (SRH) services and reproductive rights are also for all people with disabilities. Individuals with disabilities are a distinctive group, and factors such as sex, age, gender identity, sexual orientation, religion, race, ethnicity, and socioeconomic status affect their experiences in life and their health needs (Kim, Park &Namkung 2024; Wiesel et al. 2024).

Women with disabilities have the same needs for SRH as women without disabilities. In some instances, they are faced with physical, social, and attitudinal barriers in accessing community resources such as healthcare facilities, especially services of SRH (Rade et al. 2023). Global evidence suggests that women with disabilities are faced with many challenges when accessing maternal health care. They are facing unique forms of stigma and discrimination arising from the intersection of being women, mothers, and people with disabilities (Carew et al. 2024; Nguyen 2020; Nguyen, Edwards & King 2023; Shiwakoti et al. 2021).

Although women with disabilities face significant challenges during pregnancy and motherhood, many still view the experience as exceptional and deeply fulfilling (Yu, Lv & Zhang 2025). A study by Heideveld-Gerritsen et al. (2021) and Shaul, Dowling and Laden (2024) asserts that women with disabilities can fall pregnant and have the same longings to be a mother as non-disabled women. A large study of 10 718 women (US national survey of family growth) conducted from 2006 to 2010 demonstrated that women with or without disabilities have the same attitude, desires and intentions towards childbirth and motherhood (Diamanti et al. 2021).

Even though some studies look at the healthcare experiences of women with physical disabilities, there is still very little research that fully explores what pregnancy and motherhood are like for women with disabilities (Nguyen et al. 2023). Thus, this study sought to answer the following research question: ‘What are the experiences of pregnancy and motherhood among women with disabilities within Nkangala District, Mpumalanga province?’ Exploring the experiences of pregnancy and motherhood among women with disabilities is essential for informing interventions that are geared towards addressing the reproductive and sexual health needs of this population.

## Research methods and design

### Study design

A qualitative, exploratory descriptive design was used to gain an in-depth understanding of the experiences that make women with disabilities to thrive or not to thrive during pregnancy using one-on-one in-depth interviews.

### Study setting and population

The study was conducted in one of the six municipalities (Dr J.S. Moroka) in Nkangala District, Mpumalanga province. Dr J.S. Moroka is one of the largest local municipalities in Nkangala District, which offered an opportunity to access an adequate number of potential participants for this study. Dr J.S. Moroka local municipality is approximately 125.1 km northeast of Pretoria. The target population in this study was women with disabilities of reproductive age (18–45 years), who have experienced pregnancy, and/or who are mothers residing in villages during data collection.

### Inclusion and exclusion criteria

Participants with physical, visual, mental, and neurological disabilities were included. Participants with mental disabilities in this study refer to women living with mental illness who are on medication and stable and who can able to clearly describe their experiences during the interview. The study excluded women with intellectual disability, communication difficulties, and hearing difficulties.

### Sampling size

Sampling is the process or technique of selecting a suitable sample or a representative part of a population, for the purpose of determining the characteristics of the entire population (Karunarathna et al. 2024). Referred participants were recruited in the study and interviewed until data saturation was reached. The study achieved data saturation in eight interviews, and two additional interviews were done to ensure that there was no additional information emanating from the interviews. The researchers confirmed that there were no new ideas or information obtained from conversations with 10 participants (Hossain, Alam & Ali 2024; Rahimi 2024).

### Sampling technique

In this study, snowball sampling was used as the participants with the targeted characteristics were not easily found (Khoury 2024). The first two participants who met the study’s criteria were accessed from the South African Social Security Agency (SASSA) pay points with the help of the SASSA manager. After that, we asked these two participants to recommend other women with disabilities, and the potential participants were reached from their residences.

### Data collection

We collected data using an interview guide with open-ended questions. The method of data collection was one-on-one in-depth individual interviews. The interview guide was translated to IsiNdebele and Northern Sotho as they are the most spoken languages in Nkangala District. For each language translation, two independent translators who are fluent in the languages were employed to translate the tools. After the translation was done, the researchers facilitated each pair of translators to check each other’s translation and reach a consensus regarding the translation in maintaining the original meaning of questions in the tool. There were three main sections on the tool. The first section focused on the participants’ socio-demographic characteristics, the second section focused on experiences during pregnancy, and the last section focused on the experiences of motherhood.

After obtaining the study ethical clearance and permissions from the Department of Social Development, the researchers visited the offices of SASSA in Nkangala District to meet with facility manager for briefing. We presented the research purpose, the research data collection plan, and requested the space for interview in the facility. After all the logistics for data collection were addressed, we were ready to start with the recruitment.

We met with those who agreed to participate and further explained the reason for the study.

After the introductions, the researcher conducted information sessions with the participants to obtain informed consent. The participants were told that participation was voluntary and that withdrawal from the study could take place at any time without compromising the care they received from the facility. Those who consented verbally to participate in the study were taken to one of the consulting rooms reserved for data collection.

Data were collected by the primary researcher in Mmametlhake SASSA office in Dr J.S. Moroka municipality and in the houses of other participants who felt comfortable conducting the interview in their houses. Participants were interviewed every Tuesday in one of the unused offices after they had completed their visit to SASSA and others were interviewed in their homes in a room with no other people present. Each interview lasted for approximately 30–45 min. The interviews were audio-recorded with the permission of the participants. Data collection occurred from June 2024 to the end of August 2024.

### Data analysis

A voice recorder was used to record the interviews with the participants’ approval. We transcribed the audio-recorded interviews verbatim, and the transcripts were translated to English by a professional language translator. The translated transcripts were proofread in preparation for analysis. A thematic analysis approach was used, where codes are applied to text in the transcripts for the purpose of identifying patterns in the data (Özden 2024). The first six transcripts were coded by each author independently, for comparison of their application of codes for intercoder reliability. A codebook with definitions was developed and it was used to code data consistently. The transcripts were imported into Nvivo14 to organise the data and to further apply codes to the transcripts. The approach enabled us to identify patterns in the data and report the study’s findings. Socio-demographic characteristics of the study participants were summarised in a table and described narratively.

### Measures of trustworthiness

Trustworthiness or rigour of a study refers to ‘the degree of confidence in data, interpretation, and methods used to ensure the quality of a study’ (Sabnis & Wolgemuth 2024). The study’s credibility was enhanced by allowing participants to decline participation or withdraw from the study at any time. This was to ensure that data were collected from those willing to be part of the study so that they could provide the researcher with responses that truly reflected their experiences.

An audio recorder was used when conducting interviews, and probes were used to encourage participants to give more details of their experiences. The researcher ensured dependability by providing detailed information about the methods used to collect data for the study. This allowed the readers to understand the methodology and repeat the study. Transferability was ensured by providing details about the study setting so that other researchers could decide if the findings of the current study could apply in other circumstances. The researcher ensured confirmability by providing information based on the responses of the participants.

### Bias

Interview bias refers to how the interviewers’ characteristics, questioning style, and responses can influence participants’ answers (ed. Frey 2018). It is different from bias, which comes from the content or wording of questions. The researcher minimised interview bias by ensuring that she remained neutral when conducting interviews and not giving verbal or non-verbal cues. The participants’ answers were recorded and written verbatim.

The researcher reduced respondent bias by maintaining a neutral facial expression to avoid making participants feel uncomfortable. They were asked to respond honestly and told that their responses would be kept confidential and anonymous so that they could give sufficient information. Interviews were conducted in a language that the participants understood to avoid confusion and misinterpretation of the questions.

### Ethical considerations

The researcher sought ethical clearance from the Sefako Makgatho Health Sciences University Research Ethics Committee. Upon receiving a clearance certificate (SMUREC/H/389/2023:PG), a letter was sent to the Research Ethics Committee of Mpumalanga Provincial Department of Health, Nkangala District (Department of Health) and the managers of the SASSA office in Mmametlhake of Dr J.S. Moroka Municipality, asking for permission to conduct a study. Informed consent was obtained by giving participants an information sheet and a consent form to sign, upon agreeing to participate in the study. The researcher also explained the purpose of the study so that the participants could decide whether they wished to participate.

The researcher ensured confidentiality by not discussing the participants’ experiences with other people. Pseudonyms were used when analysing the data, and the document containing information gathered during data collection is protected by a password, saved on a laptop and stored for future reference. The researcher explained to the participants that there is no financial gain in agreeing to be part of the research and no favours would be given regarding the services provided in the SASSA office.

## Results

According to the demographic profile, four participants were aged 19–28 years, another four were aged 31–37 years, and two were aged 42–44 years. Six participants were born with a disability and four became disabled between 2010 and 2024. Among the study participants, four had a physical disability, three had a mental disability and the other three had a visual disability.

During data collection, seven participants were unemployed, one was doing an internship and two were employed. There were two participants with tertiary qualifications, five attended school to high school level and three attended primary school only. Three participants were married, while seven participants were single. All the participants had children: two of them had one child, five had two children, two had three children and only one had four children.

Table 1 shows the demographic characteristics of women living with disability who have fallen pregnant and are mothers.

**TABLE 1:** Socio-demographic characteristics.

Participant number	Age (years)	Born with disability Yes/No	Type of disability	Highest level of education	Occupation status	Number of children	Marital status
P1	31	No	Mental	Grade 11	Unemployed	2	Single
P2	28	Yes	Mental	Grade 7	Unemployed	3	Single
P3	20	No	Physical	Grade 12	Unemployed	1	Single
P4	26	No	Physical	Grade 11	Unemployed	2	Single
P5	44	Yes	Visual	Grade 11	Unemployed	2	Married
P6	19	Yes	Visual	Grade 7	Unemployed	2	Single
P7	37	Yes	Physical	Grade 11	Internship	1	Single
P8	34	Yes	Mental	Grade 7	Unemployed	3	Single
P9	35	Yes	Visual	Bachelor’s degree	Employed	2	Married
P10	42	No	Physical	Honours degree	Employed	4	Married

### Presenting the themes

In this study, seven themes emerged: unplanned pregnancy; mixed emotions and a sense of relief about pregnancy; termination of pregnancy; receiving support during pregnancy and after giving birth; attitude from people and health care professionals; difficulty with fulfilling motherly roles; and raising a baby while living with disability. In addition, Table 2 provides further definitions of the themes that emerged during data analysis.

**TABLE 2:** Themes and definitions.

Themes	Definition of themes
Unplanned pregnancy	Pregnancy being unplanned as mentioned by the participants.
Having mixed emotions and a sense of relief about the pregnancy	Any mentioning of having mixed emotions and a sense of relief on realising ability to conceive irrespective of their condition.
Wished to terminate the pregnancy	Any mentioning of having had a wish to terminate the pregnancy.
Receiving support during pregnancy and after giving birth	Any mentioning of having received support during pregnancy by people in their lives, and this includes support from parents, aunts, husbands, and partners.
Negative attitude from people and health care professionals about the pregnancy	Any mentioning of negative attitude about the pregnancy by the family and community members because of the participants’ disability.
Difficulty with fulfilling motherly roles	Any mentioning of difficulty to physically care for the baby because of the use of mobility assistive devices such as wheelchairs and crutches.
Raising a child while living with disability	Any mentioning of the experience of raising a child while living with a disability.

#### Theme 1: Unplanned pregnancy

Four participants reported that falling pregnant was not planned and two participants reported that they did not think that they could fall pregnant as doctors told them that because of their disability they could not conceive. All the participants verbalised that pregnancy has changed how they view other disabled women when they are pregnant. The following are some quotations raised by the participants regarding falling pregnant:

‘My pregnancy was not planned; it was a mistake.’ (P2)

‘I did not plan to fall pregnant, and I did not think I would ever be pregnant as my doctor told me that the accident damaged my tubes.’ (P3)

‘I was not planning to fall pregnant because when I was hospitalised after the accident the doctor told me that I will never walk again and I will never conceive, therefore I was not using contraceptives because I did not think that I will fall pregnant.’ (P4)

‘I was still young, and I did not think that I will fall pregnant, I thought I will work first before falling pregnant.’ (P6)

Two other participants reported that they conceived through rape, and they did not love their babies; however, as time went by, they started loving their babies. They also reported that the perpetrators were not jailed, and they have forgiven them.

#### Theme 2: Having mixed emotions and a sense of relief about the pregnancy

Four participants expressed that falling pregnant caused them to have mixed emotions and a sense of relief because the doctors told them that they would never have children because of their disabilities. They reported that falling pregnant made them feel like other women:

‘When I found out that I am pregnant I was shocked at first as I was told that I will never conceive, however as the time went by I felt relieved that I was able to conceive just like any other women.’ (P3)

‘I did not know that I am pregnant, but my partner kept on telling me that I am pregnant. When I did a pregnancy test it came out positive and I was so scared, I asked myself so many questions however as the time went by I accepted the pregnancy, and I was relieved as I was told that I will never conceive.’ (P4)

‘Pregnancy was the last thing on my mind; I was shocked and happy at the same time.’ (P8)

‘After I was told that I will never walk again, I told myself that I will not have any more children as I had three children before the accident. I had mixed emotions upon finding out that I am pregnant because I thought it was going to be difficult to take care of a baby in my condition.’ (P10)

#### Theme 3: Wished to terminate the pregnancy

Two participants who have had babies before, reported that they wanted to terminate their pregnancies:

‘I was not happy about the pregnancy I even told my husband that I do not like what is happening to me. I did not know what to do, I even suggested that I terminate the pregnancy however my husband said no. I ended up accepting the pregnancy.’ (P5)

When I found out that I am pregnant, I went to the hospital because I wanted to terminate the pregnancy, but I was told that I have passed the termination period.’ (P1)

Four other participants mentioned that their family members were the ones who suggested termination of the pregnancy to them. Although termination was suggested, none of the participants did it:

‘I did not tell anyone that I am pregnant, my sister noticed that I am pregnant when I started showing and she said I should go and terminate the pregnancy as I am bringing a burden to the family.’ (P8)

‘My dad was happy that I am pregnant, my aunt was surprised as I was told that I will never fall pregnant and my stepmother was not happy and suggested that I should terminate the pregnancy, but my dad refused.’ (P4)

‘The sister of the person who raped me noticed that I was pregnant and forced me to drink abortion pills, I was taken to seven different hospitals, and the nurses turned me back due to my disability. After failing to do the abortion at a healthcare facility, the sister of the culprit tried to do it at home by asking people to jump on top of my belly bump, it was still unsuccessful and when I gave birth my baby came out holding abortion pills.’ (P7)

‘My family did not care about me; my aunt advised me to terminate the pregnancy however my other aunt told me not to do it. As time went by I accepted the pregnancy.’ (P2)

#### Theme 4: Receiving support during pregnancy and after giving birth

Five participants indicated that they had various support structures and people who assisted them during the pregnancy and after giving birth. These participants were supported by their grandmothers, mothers, partners, neighbours, and aunts. The following statements illustrate the support received by the participants:

‘My mom took care of me when I was pregnant, she used to make food for me and help me to bathe, she continued taking care of me and the baby after giving birth. My partner used to accompany me to the clinic as I am unable to walk while holding the baby because I am using two crutches.’ (P3)

‘My dad was incredibly supportive during my pregnancy and after giving birth. My partner used to accompany me to the clinic all the time. I have received so much support even from my partner’s family, my daughter is now staying with her grandmother.’ (P4)

‘My husband supported me so much, he used to accompany me to the clinic, and he was so hands on with the baby. My mother and my brother also assisted me with the baby after she was born and at times my mother would take her to the clinic, and I will get to rest a bit.’ (P5)

‘My family supported me throughout my pregnancy; they took care of me and did not want me to work hard. After giving birth my mother assisted me with the baby for the first three months, and she went back home. My nanny made things easy for me as she was assisting with taking care of the baby and she used to take her to the clinic.’ (P9)

‘I have a nanny who helped me so much with my baby and my husband was always available to assist with the baby where he can.’ (P10)

Four other participants reported that they did not receive any support from their family members during pregnancy and after giving birth. This could have been due to their family members being against their pregnancies as they deemed them not fit to have babies while living with disabilities. Although these participants did not receive any support from their family members, they reported being able to manage to care for their babies; however, at times it was hard. One participant reported that her boyfriend was the only person who assisted her.

#### Theme 5: Attitude from people and health care professionals

Women with disabilities face barriers when it comes to their fertility aspirations and motherhood. One participant reported that she experienced positive attitudes, and three other participants experienced negative attitudes from people when they noticed their pregnancy. This is alluded by the participants by the following excerpts:

‘My neighbour used to take care of me when I was pregnant and he was happy that I did not terminate the pregnancy, however my family used to shout at me and say I am mentally ill, yet I fell pregnant.’ (P1)

‘Some of my family members and people from the society used to say why did I fall pregnant in my condition, how am I going to carry my baby while using two crutches.’ (P3)

‘My friend used to gossip about me and say I am using a wheelchair and pregnant, how am I going to take care of the baby. I was hurt by that statement.’ (P4)

‘My mother once told me that some lady was gossiping about me saying I have a toddler and now I am pregnant again while I am blind and my parents are blind.’ (P6)

‘People have negative comments when they see a disabled woman pregnant, they like saying we love men.’ (P7)

‘My neighbour asked me why I fell pregnant while I am a burden to my family.’ (P8)

Furthermore, two participants reported that they experienced negative attitudes from health care professionals when attending antenatal clinics and giving birth:

‘When visiting the clinic, the nurses used to say I fell pregnant while I am blind, what is my problem? The nurses did not treat me with respect; they used to make me want to fall pregnant while I am blind is a taboo.’ (P5)

‘When I went for my antenatal check-ups the nurses used to tell me that I did a good thing by falling pregnant, they were happy for me.’ (P10)

#### Theme 6: Difficulty with fulfilling motherly roles

Although three participants indicated they were happy to be pregnant, they reported challenges when fulfilling maternal roles especially because they are using assistive devices to walk. Some excerpts in this regard are as follows:

‘I am using two crutches to walk therefore it was difficult to carry my baby; I had to sit down if I wanted to carry him, bathe him and feed him. Taking him to the clinic on my own was also difficult because I could not even push his stroller.’ (P3)

‘I started experiencing challenges as my baby was growing, she started being heavy and I could not carry her, bathe her nor dress her because I am using a wheelchair.’ (P4)

‘Using a wheelchair made me not to be able to play with my baby up to my full satisfaction.’ (P10)

Three other participants indicated that, at times, it was difficult to take care of the baby, and they relied on their family members and/or partners for assistance. Their families would help them with taking care of the baby such as bathing them, feeding them, taking them to the clinic, and doing their laundry.

#### Theme 7: Raising a child while living with disability

All the participants had different experiences of raising their children while living with disabilities. However, seven participants reported they had challenges and needed assistance to care for their children. Regardless of the challenges, they reported that their children gave them a sense of joy and fulfilment:

‘Being a mother is nice, and I enjoy it. When my baby was born it was a challenge taking care of him because my family used to make things difficult for me and at times they would hide food from us. I take my children as the greatest blessing because now the eldest one assists me with house chores.’ (P1)

‘Motherhood is amazing, I look at my baby and feel blessed. My disability did not hinder me from being a mother. My husband and my nanny helped me a lot in raising my baby.’ (P10)

The participants reported that motherhood is challenging, especially when living with a disability because you cannot do everything that you want for the baby; they have to rely on their family members to help them. One participant reported that she is no longer staying with her daughter due to the inability to fulfil all the maternal roles. She reported that it is heartbreaking that she cannot stay with her daughter due to her condition. Three other participants reported that they did not encounter any challenges as they were able to do things for their children without needing any assistance.

## Discussion

This study explored the experiences of women living with disabilities regarding their pregnancies and motherhood. This discussion focused on the themes emerged during data analysis. Participants in this study were initially shocked when they found out they were pregnant. Participants reported that they did not plan to become pregnant, while others reported that physicians told them that women with disabilities have difficulty conceiving and giving birth due to the nature of disabilities. The association between disability and infertility remains uncertain, as few studies have examined the risk of infertility in women with and without disabilities (Ha & Martinez 2021). But the research from the 2011–2015 National Family Growth Survey shows that women with disabilities experience a significant decrease in fertility rates (Ha & Martinez 2021). Advances in reproductive healthcare have increased the desire and intention of women with disabilities to have children; however, they remain at a higher risk of adverse pregnancy outcomes (Tarasoff et al. 2020). Although the results of this study revealed shock among participants because of their belief that they could not conceive, they felt happy and relieved that they could have children like other women without disabilities and thus gave them a feeling of fulfilment.

The study reported that some participants became pregnant because they were sexually abused, which meant that pregnancy was not desired or planned. This is supported by literature where it was identified that an estimated 39.9% of women with disabilities had experienced sexual abuse, and that they were four times more likely to be abused throughout their lives (Ledingham, Wright & Mitra 2022). Although this study did not investigate why they were sexually assaulted, and the reason why persons with disabilities were at greater risk of sexual abuse, literature suggests that it might be because of an increased perceived vulnerability, decreased knowledge of sexual education, especially on sexual safety and sexual rights (Chopin, Beauregard & Deslauriers-Varin 2024; Gülay & Eratay 2024; Ledingham et al. 2022).

The findings of this study also indicate that participants had mixed emotions and a sense of relief about pregnancy. They stated that being pregnant while living with a disability shocked them as they thought they could not conceive. In turn, they felt fulfilled by knowing that they were able to conceive and taking care of their children like other women. This is supported by Morais, Moreira, and Costa (2024), who found that people with disabilities recognise the right of women with disabilities to motherhood and believe they should have the same sexual and reproductive rights as others, including the right to preserve fertility.

While some participants reported a sense of relief when they found out that they were pregnant, others reported that they wanted to terminate the pregnancy. Some reported that their family members tried to persuade them to terminate the pregnancy; however, none of the participants terminated their pregnancy. This is in agreement with the findings of a study conducted by Shpigelman and Bar (2023) where pregnant women with disabilities in this study reported that were strongly urged by their families and healthcare providers to have abortions and avoid conceiving in future. Autonomy over reproductive choices requires a combination of knowledge, access to healthcare, understanding of consent, and the ability to communicate decisions with others. On the contrary, systems and services must actively provide women with disabilities with a means of making reproductive health decisions and decisions about pregnancy and motherhood (Vallury, Tucker & Sheeran 2025). Participants in this study reported that, although they were convinced to end pregnancy, they exercised reproductive rights, not to end pregnancy, but to prepare for childbirth and motherhood.

Participants in this study highlighted various forms of support received during pregnancy and birth. They reported to have received support from family members, partners, and neighbours during and after birth. This is supported by the study by Commodari La Rosa and Nania (2022) which indicated that mothers living with disabilities received adequate support from family and friends during pregnancy and after giving birth. However, some of the women in the study reported that they went through a pregnancy alone and raised their children without any help, rather than being shouted at for being pregnant while living with disabilities. Women with disabilities face numerous obstacles to achieving sexual health, including stigma related to disability and sexuality (Carew et al. 2024).

Although rights of disabled people have improved in recent years, they continue to suffer many hardships including being stigmatised (Lanzarotti et al. 2024). Stigma affects disabled women during pregnancy, particularly through the components of social beliefs, stereotypes, and attitudes. People living with disabilities are therefore vulnerable to a lack of self-confidence and self-efficacy and to an increase in depression, abuse, neglect, exclusion, and exploitation. On the other hand, stigma influences the acceptance of cultural and social norms and creates misunderstandings that can limit women’s ability to access reproductive health services (Carew et al. 2024; Lanzarotti et al. 2024).

Another interesting finding concerns the attitudes of people and health care professionals towards women living with disabilities during pregnancy. Participants highlighted that they experienced both positive and negative attitudes. The negative attitudes outweigh the positives, as people made comments that suggest that these women are incapable of taking care of a baby because of their disabilities. In many cases, nurses are often skeptical about the ability of women with disabilities to become pregnant and care for children. As a result, they tend to focus more on the disability itself than on the women’s maternal capacities during pregnancy and postpartum care (Commodari et al. 2022). As women with disabilities are increasing and more become pregnant, it is crucial to ensure that they receive acceptable and high-quality maternity care (Blair et al. 2022).

This study found that some participants found it difficult to take care of their children. However, they had to rely on family or partner’s help. Due to their disability, they found it difficult to carry a baby while walking with crutches or using wheelchair. Some said that they depended on their family members to bath and feed their children. However, some participants reported that they were able to do everything for their children. A study by Shpigelman and Bar (2023) revealed that adults with disabilities perceive support from family members as excessive protection, affecting their ability to make their own decisions in various areas of parenting.

Furthermore, the same study found that women with disabilities felt that when family members assisted with childcare, it undermined their experience as mothers, leading to increased frustration and reduced self-efficacy and self-esteem (Shpigelman & Bar 2023). Little is known about the positive experience of mothers with disabilities. (Heideveld-Gerritsen et al. 2021). Motherhood is generally regarded as an essential element of women’s identity, and disabled mothers suggest that family support along the pregnancy, delivery, and parenting journey can help in raising their children in the community space (Shpigelman & Bar 2023).

### Implications of the study

Women with disabilities have the rights of motherhood and pregnancy. However, it is not always recognised as a right, especially by family and society. Women with disabilities deserve the same respect and dignity as non-disabled women without questioning their choices in sexual and reproductive life. The maternal and childbirth experiences of this group are often overlooked and rarely discussed. Therefore, healthcare providers should prioritise this issue to raise awareness among women living with disabilities.

The lack of support during pregnancy and after birth has serious impacts on disabled women; therefore, emotional and psychological support is needed in this population. This is particularly important in rural areas, where communities are often unaware of the reproductive health needs for women with disabilities. Raising awareness can help reduce the stigma and discrimination they face during pregnancy with disabilities can also have normal babies, which means that people should not be judgemental. Furthermore, depending on the severity of the disability, these women can still meet the needs of their children, while other women need minimal to moderate support. Women with disabilities are exposed to negative attitudes in society and by health professionals. Therefore, more research is needed to focus on intervention and implementation of solutions to address or reduce negative attitudes towards women living with disabilities during pregnancy and child-rearing.

### Limitations of the study

This study has certain limitations. As it was conducted in a single rural district, the findings may not be transferable to women with disabilities living in urban settings or other regions with diverse cultural or healthcare contexts. The study relied on participants’ self-reported experiences, which may be influenced by recall or social desirability bias. However, efforts were made to ensure credibility through prolonged engagement with participants, triangulation of data sources, and verbatim transcription. Future studies could explore similar experiences in diverse settings to enhance the transferability of findings.

### Recommendations

Many women with disabilities have limited knowledge of reproductive health. Healthcare providers should prioritise this area and strengthen their capacity to offer inclusive antenatal and postnatal care. It is recommended that programmes supporting women with disabilities during pregnancy and after birth should be strengthened to raise awareness of society in general, and policymakers should protect women with disabilities during pregnancy so that they are not stigmatised and rejected. Women with disabilities face the challenges of mental health more in pregnancy and childbirth, and it is important to also take into account their mental health status during maternal care. Women with disabilities must also express their challenges and situations so that needed support can be provided.

## Conclusion

The key findings of the study indicate that the women interviewed did not plan to become pregnant, as they felt their disabilities prevented them from being allowed to have children. However most have welcomed and accepted the pregnancy and the status of being mothers. The data show that when participants realised that they were pregnant, they were initially shocked, which changed their perspective on women with disabilities. The results show that women with disabilities have been given adequate support during pregnancy, birth and childcare. However, the other participants did not receive any support but were able to care for their children and even ignored the call to end pregnancy.

Some women have reported sexual abuse, which has led to pregnancy. Furthermore, the study showed that participants faced negative attitudes from community members, and nurses felt that they had made a mistake by being pregnant while living with disabilities. Participants highlighted some difficulties in raising children. However, they also reported that being a mother is fulfilling their desire to be parents even though they are living with disabilities.
